# Supplementary material for: Mapping the evolution of fertility support policies in China: A content and instrumental analysis
Source: PLoS One. 2025 Oct 9;20(10):e0332137. doi: 10.1371/journal.pone.0332137 (PMC12510515; doi:10.1371/journal.pone.0332137)
Supplement: S1 Appendix — (ZIP) [file pone.0332137.s001.zip › S1 Appendix. 226 original policy documents/191-国家发展改革委、中央宣传部、教育部等关于印发《加大力度推动社会领域公共服务补短板强弱项提质量 促进形成强大国内市场的行动方案》的通知(FBM-CLI-4-329620).doc]

国家发展改革委、中央宣传部、教育部等关于印发《加大力度推动社会领域公共服务补短板强弱项提质量 促进形成强大国内市场的行动方案》的通知

**发布部门：** [国家发展和改革委员会(含原国家发展计划委员会、原国家计划委员会)](https://www.pkulaw.com/law/chl?Aggs.IssueDepartment=60301) [中共中央宣传部](https://www.pkulaw.com/law/chl?Aggs.IssueDepartment=a04) [教育部](https://www.pkulaw.com/law/chl?Aggs.IssueDepartment=60203) [机构沿革](javascript:void(0))

**发文字号：**发改社会〔2019〕0160号

**发布日期：**2019.01.23

**实施日期：**2019.01.23

**时效性：** [现行有效](https://www.pkulaw.com/law/chl?Aggs.TimelinessDic=01)

**效力级别：** [部门规范性文件](https://www.pkulaw.com/law/chl?Aggs.EffectivenessDic=XE0302)

**法规类别：** [市场管理](https://www.pkulaw.com/law/chl?Aggs.Category=08204) [营商环境优化](https://dobusiness.pkulaw.com/)

国家发展改革委、中央宣传部、教育部、工业和信息化部、民政部、财政部、人力资源社会保障部、自然资源部、住房城乡建设部、农业农村部、商务部、文化和旅游部、国家卫生健康委、广电总局、体育总局、国家文物局、国家中医药局、中国残联关于印发《加大力度推动社会领域公共服务补短板强弱项提质量 促进形成强大国内市场的行动方案》的通知
（发改社会〔2019〕0160号）

各省、自治区、直辖市、新疆生产建设兵团有关部门、机构 ：
　　现将《加大力度推动社会领域公共服务补短板强弱项提质量 促进形成强大国内市场的行动方案》印发给你们，请认真贯彻执行。

国家发展改革委
中央宣传部
教　育　部
工业和信息化部
民　政　部
财　政　部
人力资源社会保障部
自然资源部
住房城乡建设部
农业农村部
商　务　部
文化和旅游部
国家卫生健康委
广 电 总 局
体 育 总 局
国家文物局
国家中医药局
中 国 残 联
2019年1月23日

　　加大力度推动社会领域公共服务补短板强弱项提质量促进形成强大国内市场的行动方案

　　社会领域公共服务关乎民生，连接民心。补齐社会领域基本公共服务短板，增强非基本公共服务弱项，提升公共服务质量和水平，既有利于加强社会政策兜底保障、在发展中保障和改善民生，又有助于扩大公共服务有效供给、积极培育发展国内市场，对于新时代满足人民日益增长的美好生活需要、高水平全面建成小康社会、促进社会公平正义都具有十分重要的意义。
　　近年来，社会领域公共服务投入不断加大，设施条件不断改善，但相对于群众多层次多样化需求，仍然存在供给不足、质量不高、发展不均衡等突出问题，托幼、上学、就医、养老等方面的服务质量和水平与群众期待还有不小差距，这既是现阶段社会主要矛盾的表现，又是供给侧结构性改革的重点，也是潜在的巨大国内市场。为加大力度推动社会领域公共服务补短板、强弱项、提质量，促进形成强大国内市场，特制定以下方案。
　　**一、**总体要求
　　（一）指导思想。
　　以习近平新时代中国特色社会主义思想为指导，全面贯彻落实党的十九大和十九届二中、三中全会精神，坚持新发展理念和以人民为中心的发展思想，按照高质量发展要求，对接脱贫攻坚、乡村振兴、科教兴国、健康中国、创新驱动等国家战略，贯彻“巩固、增强、提升、畅通”八字方针，深入推进公共服务供给侧结构性改革，牢牢抓住人民群众最关心最直接最现实的利益问题，正确处理基本与非基本、政府与市场、供给与需求的关系，着力补齐基本公共服务短板、增强非基本公共服务弱项、提升公共服务质量水平，切实兜牢基本民生保障网底，不断满足多样化民生需求，努力增进全体人民在共建共享发展中的获得感、幸福感、安全感，让改革发展成果更多更公平惠及全体人民。
　　（二）基本原则。
　　--补齐短板，保障基本。采取针对性更强、覆盖面更广、作用更直接、效果更明显的举措，优先补齐基本公共服务短板，促进公共服务资源向基层延伸、向农村覆盖、向边远地区和生活困难群众倾斜，推进基本公共服务均等化、普惠化、便捷化。
　　--补强弱项，扩大供给。政府在做好政策制定、规划引领、环境营造、监管服务的前提下，充分发挥市场和行业协会、商会等社会组织的作用，鼓励引导社会力量参与，扩大公共服务有效供给，推动非基本公共服务市场化、多元化、优质化。
　　--创新机制，提升质量。破除体制障碍，创新提供方式，优化资源配置，加强人才培养，加快与互联网深度融合，推进城乡区域基本公共服务制度统一，推动优质服务资源延伸下沉和提质扩容，促进公共服务更高质量更高水平发展。
　　--尽力而为，量力而行。将提升保障和改善民生水平建立在经济和财力可持续基础上，既要加大保障和改善民生工作力度，又要合理引导社会预期，不吊高胃口，不过度承诺，确保公共服务水平在发展中不断完善、稳步提升。
　　（三）主要目标。
　　到2020年，公共服务供给结构更加合理，社会力量参与更加积极，实施保障机制更加完善，服务质量与水平不断提升，公共服务对保障民生、促进就业、扩大消费的作用不断增强，基本实现基本公共服务能力全覆盖、质量全达标、标准全落实、保障应担尽担，实现非基本公共服务付费可享有、价格可承受、质量有保障、安全有监管。其中，教育现代化取得重要进展，劳动年龄人口平均受教育年限达到10.8年；覆盖城乡的基本医疗卫生制度基本建立，人均预期寿命提高到77.3岁；以居家为基础、社区为依托、机构为补充、医养相结合的养老服务体系更加完善，养老床位中护理型床位比例不低于30%；婴幼儿照护服务的政策法规和标准规范体系初步建立；现代公共文化服务体系基本建成，文化产业成为国民经济支柱性产业；旅游经济稳步增长，对国民经济的综合贡献度达到12%；群众身体素质稳步增强，人均体育场地面积达到1.8平方米；家政培训标准化程度进一步提升，行业规范化建设进一步巩固。
　　到2022年，公共服务供给更加充足、资源布局不断优化、体制机制日趋完备、人才队伍发展壮大、服务质量明显提高，覆盖全民、普惠共享、城乡一体的基本公共服务体系不断健全，就近就便、高效快捷、便民利民的公共服务体验不断改善，政府保障基本、社会积极参与、全民共建共享的公共服务格局不断完善，社会关注的民生热点难点问题得到有效缓解，多样化可选择的公共服务资源更加丰富，潜力巨大的国内市场需求得到满足，广大群众的获得感、幸福感、安全感不断提升。

　　**二、**行动任务
　　（一）补齐基本公共服务短板，加快实现基本公共服务均等化。
　　1.推进义务教育均衡发展。推进县域内城乡义务教育学校建设标准统一、教师编制标准统一、生均公用经费基准定额统一、基本装备配置标准统一和“两免一补”政策城乡全覆盖。继续加大对困难地区和薄弱学校支持力度，完善农村小规模学校（教学点）基本办学条件，落实对不足100人的小规模学校按100人标准拨付公用经费。加强乡镇寄宿制学校建设，为住宿学生配备宿舍、食堂、厕所、澡堂等基本生活设施，落实对寄宿制学校按寄宿生年生均200元标准增加公用经费补助。合理有序扩大县镇学校学位供给，到2020年基本消除大班额。加强学校非卫生厕所改造。巩固基本均衡发展成果，稳步推进县域义务教育优质均衡发展，到2022年全面实现全国县域义务教育基本均衡发展，约20%的县（市、区）实现义务教育优质均衡发展。
　　2.提升贫困地区县域医疗卫生服务能力。改善农村医疗卫生保障条件，确保每个县（市、区）建好1-2所县级公立医院（含中医院），并以集中连片特殊困难地区和国家扶贫开发工作重点县为重点，改善基础设施和装备条件。力争到2020年，每千人口县级医疗机构床位数达到1.8张左右，能够满足县域居民常见病、多发病诊疗等需要。地方政府负责筹集资金，加强社区卫生服务中心（站）、乡镇卫生院和村卫生室的基础设施标准化建设。推动基层医疗卫生机构提供中医特色服务，充分发挥中医药“简、便、验、廉”优势。
　　3.加强妇幼健康服务体系建设。重点支持床位不足或业务用房面积不达标的妇幼保健机构建设，力争省、地市、县三级都有一所政府举办、设施齐全的妇幼保健机构，强化省、地市、县三级危重孕产妇、新生儿救治能力和产前诊断筛查能力。支持儿科资源不足地区加强建设，统筹利用各种服务资源，鼓励有条件的妇幼保健机构扩展强化产科、儿科、中医科等服务功能，力争到2020年每千名儿童床位数增加到2.2张。
　　4.提高医学应急救援和传染病等防治能力。依托大型综合性医院扩展补充紧急医学救援能力，配置医学救援设施设备，建立国家综合、航空、海（水）上紧急医学救援基地，构建全国分区域布局的海陆空立体化、专业化紧急医学救援网络，建立健全紧急医学救援体系。改革和完善疾病预防控制体系，加强综合性医院传染病、精神病诊疗能力和传染病医院建设，全面提升艾滋病、结核病等重大传染疾病防治能力。完善血站服务体系，加强血站及血液筛查实验室建设，保障血液安全供应。
　　5.健全基本养老服务体系。继续加强公办养老机构建设，落实新建城区、居住（小）区按照人均不少于0.1平方米的标准配建养老服务设施，稳步提高护理型床位占比。推进公办养老机构入住综合评估制度，优先满足失能老年人的基本养老服务需求。实施特困人员供养服务设施和服务质量达标工程，提升特困人员供养服务机构托底保障能力。鼓励有条件的农村特困供养服务机构（农村敬老院），在满足特困人员集中供养需求的前提下，逐步为农村低保、低收入家庭和建档立卡贫困家庭中的老年人，提供低偿或无偿的集中托养服务。大力发展农村互助养老服务，统筹规划建设公益性养老服务设施，推动50%的乡镇建有1所农村养老服务机构。
　　6.加强社会福利服务体系建设。在人口50万以上或孤儿200个以上的县（市、区）建设儿童福利机构和设施，为各级儿童福利机构配套“养治教康”方面服务设施，推动形成功能完备、服务规范的儿童福利服务设施网络。在各县（市、区）新建或依托现有资源建设县级未成年人保护设施，为困境儿童、农村留守儿童等提供临时监护照料。在精神卫生服务能力不足且尚无精神卫生福利设施的地市建设1所精神卫生福利设施，为精神障碍患者提供集中养护服务。在火葬区尚无设施的县（市、区）新建殡仪馆或公益性骨灰安放设施，对已达危房标准、设施设备陈旧的县（市、区）殡仪馆实施改扩建，对已达到强制报废年限或不符合国家环境保护标准的县（市、区）火化设备进行更新改造。
　　7.提升公共就业创业服务水平。推进全方位公共就业服务，建立全国统一的公共就业创业服务平台，加大对贫困地区、农村地区公共就业创业服务倾斜力度。实施基层公共就业创业服务能力保障
　　计划，开展公共就业服务人员能力提升专项培训，健全均等化、专业化、智能化的服务体系。制定实施公共就业创业服务设施、设备配置、人员配备、服务规范等指导性标准，统一公共就业创业服务体系视觉识别系统。加快推动公共就业创业服务数字化转型，打造集政策解读推送、业务办理咨询于一体的线上智能服务、线下自助服务体系。
　　8.推动基本公共文化服务均等化。推动落实国家基本公共文化服务指导标准和省级实施标准，以县为单位实施。推动重点文化惠民工程资源整合和改革创新，加强广播电视传输覆盖等基础设施建设，推进数字广播电视户户通和应急广播体系建设，强化数字文化服务和流动文化服务，不断提高文化惠民工程的覆盖面和实施效果。继续支持全国爱国主义教育示范基地、博物馆、美术馆、公共图书馆、文化馆（站）等公共文化设施免费开放，促进高校博物馆、美术馆等文化设施向社会开放。充分利用现有城乡公共设施，统筹建设基层综合文化服务中心。以县级文化馆、图书馆为中心推进总分馆制建设，加强对农家书屋、农村电影放映工程的统筹管理，实现城乡社区公共文化服务资源整合和互联互通。
　　9.推动公共体育设施建设和开放。继续加强县级公共体育场、全民健身中心、健身步道、体育健身公园、农民体育健身工程、社区多功能运动场、社会足球场等公共体育设施建设。合理利用公园、体育中心、闲置厂房、校舍操场、社区空置场所等，拓展公共体育设施场所。进一步完善管理机制，提升各类公共体育场馆免费或低
　　收费开放服务水平，促进学校体育设施与公共体育设施的双向开放。
　　10.健全完善残疾人公共服务体系。全面实施残疾儿童康复救助制度，有条件的地方可以对城乡贫困残疾人、重度残疾人基本型康复辅助器具配置给予补贴，实施残疾人精准康复服务行动，推进康复大学筹建。发展重度贫困残疾人托养照料服务，加快制定残疾人托养、康复服务国家标准，加强对孤残儿童福利和流浪乞讨残疾人救助服务的支持力度，加大严重精神障碍患者救治帮扶力度，对一户多残、以老养残等特殊困难家庭予以帮扶。采取普通学校就读、特教学校就读、儿童福利机构特教班就读、送教上门等方式，落实“一人一案”，解决未入学残疾儿童少年义务教育问题。完善困难残疾人生活补贴和重度残疾人护理补贴制度，推动建立补贴标准动态调整机制。结合实施易地扶贫搬迁和农村危房改造工程，加大重度贫困残疾人家庭无障碍改造力度。
　　（二）补强非基本公共服务弱项，着力增强人民群众公共服务供给。
　　11.增加托育服务有效供给。制定行业准入标准、管理规范和监管标准，明确婴幼儿照护服务对象、服务内容、从业要求、设施设备、技术流程等规范标准。充分调动各方面力量，在城市建成一批示范性婴幼儿照护服务机构，在农村和贫困地区进一步提升婴幼儿照护服务能力。利用社区中心、闲置校舍等存量资源建立婴幼儿看护中心，鼓励有条件的地方举办婴幼儿照护服务机构，提供日间照料服务。积极引导社会力量举办托育服务机构，鼓励家庭育儿知识
　　传播、社区共享平台等托育服务新模式新业态探索发展，发展多元化托育服务体系。积极开展保育知识、技能等培训，推动高职院校设置相关专业，开展家庭科学育儿培训指导。加强对儿童生长发育、儿童营养、儿童心理、儿童运动等方面的综合干预，充分开发儿童潜能，促进儿童早期发展。
　　12.扩大城乡普惠性学前教育资源。扩大公办优质学前教育资源覆盖面，充分利用各类资源发展公办园，规范城镇小区配套幼儿园建设。通过购买服务、财政奖补等多种方式支持优质普惠学前教育资源扩容。进一步完善普惠性民办园认定标准和扶持政策，通过购买服务、减免租金、综合奖补、教师交流培训等方式，支持普惠性民办园发展。到2020年，全国学前三年毛入园率达到85%，80%以上的在园幼儿享受到普惠性学前教育，本专科学前教育专业毕业生规模达到20万人以上，逐步提高公办园在园幼儿占比，全国原则上达到50%。积极支持多渠道投入学前教育。保障幼儿园教师地位待遇，落实学前教育资助制度，确保普惠性幼儿园的家庭经济困难儿童、孤儿和残疾儿童得到资助。大力发展农村学前教育，每个乡镇原则上至少办好一所公办中心园，鼓励以定向培养方式为贫困地区农村培养幼儿教师。
　　13.促进社会办医加快发展。支持社会力量举办、运营高水平全科诊所，建立专业协作团队为居民提供医疗、公共卫生、健康管理等签约服务。支持社会力量深入专科医疗等细分服务领域，在眼科、骨科、口腔、妇产、儿科、肿瘤、精神、医疗美容等专科以及中医、康复、护理、体检等领域，加快打造一批具有竞争力的品牌服务机构。鼓励有实力的社会办医疗机构瞄准医学前沿，组建优势学科团队，提供以先进医疗技术为特色的医疗服务。督促各地落实社会办医疗机构跨部门全流程综合审批指引，精简优化审批流程。
　　14.全面放开养老服务市场。到2022年，全面建成以居家为基础、社区为依托、机构为补充、医养相结合，功能完善、规模适度、覆盖城乡的养老服务体系，社区日间照料机构覆盖率大于90%，居家社区养老紧急救援系统普遍建立，“一刻钟”居家养老服务圈基本建成。推动民办养老机构发展，取消养老机构设立许可，支持境内外资本投资举办养老机构，落实同等优惠政策。深化非营利性养老机构登记制度改革，允许养老机构依法依规设立多个服务网点，实现规模化、连锁化、品牌化运营。鼓励民间资本对企业厂房、商业设施及其他可利用的社会资源进行整合和改造后用于养老服务。开展城企协同推进养老服务发展行动计划。
　　15.加强老年人健康服务体系建设。建立和完善覆盖全人群全生命周期，包括预防、保健、治疗、康复、护理、安宁疗护的综合性、连续性的服务体系。以老年人健康为中心，优化老年医疗卫生资源配置，推动医疗机构与养老机构加强合作。推广老年人健康体检，到2022年，基本实现65岁以上老年人健康体检全覆盖。加强康复医院、护理院和综合性医院老年医学科建设，老年医疗服务需求强烈的地区，可依据现有医疗机构，以多种形式加强老年专业医疗机构能力建设。推动基层医疗卫生机构积极开展老年人医疗、康复、
　　护理、家庭病床等服务，提高老年人医疗卫生服务的可及性。
　　16.推进家政培训和就业服务。持续开展家政服务提质扩容行动。实施农民工学历和能力提升行动计划、巾帼家政服务专项培训工程，推进家政培训提升行动，搭建家政服务员和用工企业对接平台，开展订单、定向培训，提升家政服务能力。全面推进“百城万村”家政扶贫，引导中西部地区贫困县与中心城市劳务对接，促进建档立卡贫困劳动力就业。建立健全家政服务国家标准、行业标准和服务规范，建设包含家政服务员基本信息、从业经历和服务技能等内容的家政服务员档案数据库和家政企业数据库。
　　17.推广城乡社区助餐服务。把社区助餐纳入国家积极应对人口老龄化中长期规划能力评价指标体系，着力解决空巢老人居家、社区养老的餐饮问题。建立健全社区助餐服务标准体系，不断提高服务效率，切实保障质量安全，丰富个性化产品，根据青年家庭、空巢老人家庭的饮食需要，推出定制化家政餐饮服务。提升餐饮企业社区助餐服务水平，鼓励餐饮企业通过电话送餐、互联网送餐等方式拓展服务范围，将服务下沉到社区，为社区居民提供生活便利。
　　18.加强优秀传统文化保护传承利用。推动大运河文化保护传承利用，加强大运河文化带、革命文物等遗产保护利用设施建设。推动文化文物单位文化创意产品开发，打造优质、特色服务品牌。编制国家传统工艺振兴目录，选取并重点支持一批传统工艺项目。开展中华优秀传统文化传承重点工程和基地建设，到2020年建设100个左右中华优秀传统文化传承基地。推进“互联网＋中华文明”行动计划，系统完整保存文物信息，推进资源开放共享。实施乡村文化繁荣兴盛重大工程，支持传统村落文物保护利用设施及配套基础设施建设，推动优秀农耕文化遗产合理适度利用。
　　19.完善重点地区旅游基础设施。实施“三区三州”等深度贫困地区旅游基础设施改造升级行动计划，切实改善当地道路、步行道、停车场、供水供电、垃圾污水处理、消防安防、应急救援、游客信息等服务设施。推动全域旅游示范区创建单位加强旅游基础设施和公共服务设施建设，着力改善景区连接道路、停车场、污水垃圾处理设施，完善集散咨询服务体系、自驾游服务体系，规范旅游引导标识系统。
　　20.加快有线电视网络整合发展和互联互通。推动完成全国有线网络整合，形成全国一张网。建立全国有线网络统一运营体系，统筹开展全国互联互通平台建设，建设国家级广电云数据中心，加大骨干网技术改造，完善接入网建设，完成双向化、宽带化、智能化改造，积极发展高清电视、超高清电视、互动电视等新业务，拓展综合信息服务和智能化应用等新业态，建设新型家庭信息中心，满足用户对于跨屏、跨域、跨网、跨终端的收视和信息需求。
　　21.加快发展体育健身休闲运动和竞赛表演业。以举办2022年冬奥会和冬残奥会为契机，大力发展冰雪运动，加强冰雪人才培养，提升群众普及水平。加强足球、冰雪、水上、航空、山地户外等健身休闲运动场地设施建设，支持城市举办马拉松、自行车等大型群众性体育赛事。加快制定赛事审批取消后的服务管理办法，明确体育培训、赛事开展的基本条件、标准、规则、程序和各环节责任部门，推动大型赛事活动多部门联合“一站式”服务。
　　（三）充分发挥有效市场和有为政府作用，提升公共服务质量水平。
　　22.提升教育服务内涵质量。建设“互联网＋教育”支撑服务平台，促进信息技术与教育教学深度融合，推动优质教育资源覆盖各类学生。提高学前教育保教质量，遵循幼儿身心发展规律，坚持科学保教，纠正幼儿园“小学化”倾向。实施义务教育质量提升工程，优化教材和课程体系，完善质量监测制度，提高义务教育巩固水平。完善职业教育和培训体系，健全德技并修、工学结合的育人机制，深化产教融合、校企合作，推动产教融合型城市遴选和企业试点，吸引企业等社会力量建设一批具有辐射带动引领作用的高水平专业化产教融合实训基地。加快推进“双一流”建设，加大东部高水平大学对中西部地区高校的支持力度，继续支持中西部地区重点建设一批高水平本科学校。
　　23.均衡发展优质医疗资源。围绕严重威胁群众健康的肿瘤、心脑血管等重点疾病，在全国建立若干高水平的国家医学中心和一批区域医疗中心，扩大优质医疗资源供给，集聚医教研产等各种创新要素，开展癌症等重大疾病科技攻关，在医学科研成果临床转化、攻克疑难复杂疾病、建立人才培养模式等领域发挥辐射带动作用。鼓励优质医疗资源通过组建专科联盟、建设分支机构、远程医疗协作、“互联网＋医疗健康”等多种方式，辐射带动中西部地区医疗服务能力提升，逐步满足群众就近享有高水平医疗服务需求。
　　24.提升养老服务质量。推进医养结合，为老年人提供患病期治疗、康复期护理、稳定期生活照料以及安宁疗护一体化的健康和养老服务。支持适合老年人的智能化产品、健康管理设备、健康养老移动应用软件（APP）等设计开发，开展智慧健康养老应用试点示范。加快建立全国统一的养老服务质量标准和评价体系，到2020年全面实施全国养老机构服务质量等级评定工作。
　　25.提高公共文化服务效能。在商场、写字楼等各类场所嵌入文化消费内容。推动公共图书馆、文化馆（站）等公共文化机构实行错时开放。引入社会化机制，以政府购买服务等方式引导更多主体参与公共文化服务，定期开展全国性公共文化产品采购大会，实现供需对接。统筹整合公共数字文化工程，加强公共文化大数据采集、存储和分析处理，建设文化云平台，推动数字图书馆、数字文化馆、智慧博物馆等公共数字文化建设项目，加强文物保护单位及可移动文物数字保护展示利用和非物质文化遗产数据库及数字展示。
　　26.加快智慧广电发展。加快大数据、云计算、人工智能等新一代信息技术在广播电视传输覆盖网中的应用，打造功能更加强大的主流媒体融合传播网、数字文化传播网、基础战略资源网，为用户提供融合新闻资讯、视听节目、社会服务、医疗健康、数字娱乐、教育培训、智能家居等多功能于一体的智慧广电数字生活服务，实现广播电视人人通、移动通、终端通。
　　27.推进多种旅游业态发展。推动乡村旅游提质扩容，完善乡村旅游服务标准，做好典型案例梳理，召开现场会推广乡村旅游发展经验。探索名城名镇名村保护与可持续发展模式，完善城市商业区旅游服务功能，建设一批避暑避寒度假目的地，打造一二三产业融合发展的美丽休闲乡村，推动建设自驾车房车旅游营地，推广精品自驾游线路。实施全国红色旅游经典景区三期总体建设方案，增强红色旅游经典景区的教育功能和脱贫富民功能。推动中医药健康旅游，建设一批中医药健康旅游示范区和示范基地。

　　**三、**组织实施
　　（一）加强统筹协调。
　　建立健全工作协调推进机制，强化部门协同、上下联动、统筹协调。各有关部门要按照职责分工，加大对本领域公共服务工作的支持力度，细化制定配套制度和政策，加强对地方的监督和指导，推动各领域重点任务、保障措施有效落实。各级人民政府要强化主体责任，明确责任分工和完成时限，优化服务流程，提高质量效率，结合实际抓好贯彻落实，并及时向上级政府和有关部门报告进展情况。
　　（二）推进项目建设。
　　各地各有关部门要瞄准重点领域投资缺口，制定涵盖基本和非基本公共服务项目实施的专项行动计划，细化社会领域公共服务短板和弱项需求测算，依托国家重大建设项目库，分近期、中期、长期进行项目储备并确保滚动接续，在项目建设过程中，严格执行各类公共服务设施建设标准。各地要切实落实现有各项产业用地政策，经相应行业主管部门认定，投资主体依法依规使用旧厂房、仓库等存量房屋提供公共服务的，可享受在五年内继续按原用途和土地权利类型使用土地的过渡期政策。
　　（三）扩大资金投入。
　　积极发挥政府投资引导带动作用，合理安排经费并及时下达资金，确保中央财政性资金优先支持基本公共服务领域补短板项目建设。鼓励地方政府依法合规采取政府和社会资本合作（PPP）等方式，吸引更多社会力量参与建设、运营和服务。加大金融支持力度，综合利用债券、保险、信贷、产业投资基金等方式，为社会领域公共服务项目融资提供支持。
　　（四）强化实施监管。
　　继续深入推进“放管服”改革，加大对储备项目的协调调度力度，加快推进项目前期工作和开工建设，推动在建项目顺利实施，确保工程质量安全，早日建成发挥效益，避免形成“半拉子”工程。各地在建设过程中，要根据财政承受能力和政府投资能力，严格项目条件审核，合理安排项目建设，严禁以政府投资基金、政府和社会资本合作（PPP）、政府购买服务等名义变相举债，防范地方政府隐性债务风险。
　　（五）加大人才培养。
　　完善人才服务体制机制，结合实际推进实施工资待遇、技术等级评价、职称评定等激励政策。健全公共服务从业人员教育培训制度，定期组织职业培训和业务轮训，提高公共服务专业化水平。充分发挥高等院校、职业学校、科研院所作用，促进专业服务和管理人才培养，引导公共服务和管理人才向中西部地区和基层流动。探索公办与非公办公共服务机构在技术和人才等方面的合作机制，对非公办机构的人才培养、培训和进修等给予支持。
　　（六）实施考核评价。
　　将公共服务机构、从业人员、服务对象诚信情况纳入信用记录，并共享至全国信用信息共享平台，同步在“信用中国”网站和国家企业信用信息系统公示，加强守信联合激励、失信联合惩戒。各有关部门要加强对地方落实本领域任务的监督检查，发展改革委将会同相关部门开展基本公共服务达标考核和城市公共服务水平评价，委托第三方开展社会满意度调查，加强事中事后监管，重大问题及时向党中央、国务院报告。
　　附件：重点任务分工方案

© 北大法宝：（[www.pkulaw.com](https://www.pkulaw.com/)) 专业提供法律信息、法学知识和法律软件领域各类解决方案。北大法宝为您提供丰富的参考资料，正式引用法规条文时请与标准文本核对。
欢迎查看所有[产品和服务](https://www.pkulaw.com/helps/2.html)。法宝快讯：[如何快速找到您需要的检索结果？](https://www.pkulaw.com/helps/70.html)[法宝](https://www.pkulaw.com/helps/69.html)V6有何新特色？

扫描二维码阅读原文

**原文链接：**<https://www.pkulaw.com/chl/7c05b1de52edfe0cbdfb.html>
